# Direct Methamphetamine Sensing in Flowing Wastewater via a 3D-Printed Flow-Through Cell

**DOI:** 10.3390/jox16020040

**Published:** 2026-02-25

**Authors:** Veronika Svitková, Ivana Horáková, Viliam Kolivoška, Eva Vaněčková, Olívia Dakošová, Eva Melníková, Dušan Žabka, Zuzana Imreová, Alexandra Tulipánová, Alexandra Paulína Drdanová, Marek Haššo, Peter Nemeček, Michal Hatala, Tomáš Mackuľak, Miroslav Gál

**Affiliations:** 1 Department of Inorganic Technology, Faculty of Chemical and Food Technology, Slovak University of Technology in Bratislava, Radlinského 9, 812 37 Bratislava, Slovakia; olivia.dakosova@stuba.sk (O.D.); eva.melnikova@stuba.sk (E.M.); miroslav.gal@stuba.sk (M.G.); 2 MicroPoll s.r.o., Vazovova 5, 812 43 Bratislava, Slovakia; zuzana.imreova@stuba.sk (Z.I.); tomas.mackulak@stuba.sk (T.M.); 3 Department of Environmental Engineering, Institute of Chemical and Environmental Engineering, Faculty of Chemical and Food Technology, Slovak University of Technology in Bratislava, Radlinského 9, 812 37 Bratislava, Slovakia; ivana.horakova@stuba.sk (I.H.); dusan.zabka@stuba.sk (D.Ž.); alexandra.tulipanova@stuba.sk (A.T.);; 4 Lapidum nigrum s.r.o., Za Obchody 841, 277 11 Neratovice, Czech Republic; viliam.kolivoska@jh-inst.cas.cz (V.K.); eva.vaneckova@jh-inst.cas.cz (E.V.); 5 Institute of Chemistry and Environmental Sciences, Faculty of Natural Sciences, University of Ss. Cyril and Methodius in Trnava, Nám. J. Herdu 2, 917 01 Trnava, Slovakia; marek.hasso@ucm.sk (M.H.); peter.nemecek@ucm.sk (P.N.); 6 Department of Graphic Arts Technology and Applied Photochemistry, Faculty of Chemical and Food Technology, Slovak University of Technology in Bratislava, Radlinského 9, 812 37 Bratislava, Slovakia; michal.hatala@stuba.sk; 7 Institute of Integral Safety, Faculty of Materials Science and Technology in Trnava, Slovak University of Technology in Bratislava, Jána Bottu, 2781/25, 917 24 Trnava, Slovakia

**Keywords:** 3D-printed flow-through cell, electrochemical sensor, illicit drugs, methamphetamine, wastewater

## Abstract

The rapid, field-ready detection of methamphetamine (MET) directly in sewage under flow remains a bottleneck for public health and law enforcement surveillance. We engineered a low-cost, 3D-printed flow-through electrochemical cell that houses a commercial screen-printed carbon electrode and operates in both non-flow and flow regimes. The platform was validated using the [Ru(NH_3_)_6_]^3+/2+^ couple, confirming negligible kinetic hindrance and suitability for voltammetric sensing under convective transport. Using square wave voltammetry and chronoamperometry, MET was quantified in filtered wastewater, with limits of detection of 15.9 µg L^−1^ in non-flow and 211.2 µg L^−1^ in flow conditions. Specificity tests yielded well-separated faradaic responses for the pre precursor α-phenylacetoacetonitrile (APAAN) and for MET, while amphetamine produced only a weak signal, enabling side-by-side discrimination in a single run. To our knowledge, this is the first demonstration of direct electrochemical sensing of MET in flowing wastewater using a 3D-printed flow-through platform. The simple, disposable design provides an actionable foundation for portable, near-real-time sewer surveillance and motivates antifouling/auto-cleaning strategies for long-term deployment.

## 1. Introduction

Methamphetamine (MET), commonly known as meth, crystal, or ice, is a drug approved by the FDA for the treatment of attention deficit hyperactivity disorder (ADHD), but also stands as one of the most prevalent and destructive illicit drugs in Europe [1,2]. The drug’s potency, affordability, and addictive properties contribute to its popularity among recreational users and individuals seeking stimulant effects. Its widespread use, coupled with its detrimental effects on both individuals and communities, has prompted significant attention from researchers, policymakers, and law enforcement agencies globally. Originating from clandestine laboratories, MET enters communities through various channels, including illicit drug markets, criminal networks, and informal economies [3,4].

The production of MET and amphetamines in general has certain specificities compared to, for example, cannabinoids and opiates, mainly in that the precursors are available (there is no need to grow a large number of plants), and the syntheses are relatively quick and simple. Precursor dynamics add critical context. α-Phenylacetoacetonitrile (APAAN), a key pre-precursor for amphetamine type stimulants, surged in seized MET samples in parts of Europe. In 2009, APAAN was nearly absent from samples, whereas by 2014, it was detected in almost 100% of collected samples [5]. Despite international regulatory efforts, APAAN continues to be widely used illicitly [6]. Simultaneous monitoring of APAAN with MET can therefore help distinguish consumption from on-site synthesis and support targeted interventions.

In Slovakia and the Czech Republic, for example, small “kitchen laboratories” used for domestic MET production underscore the value of field-compatible tools that can screen both products and precursors. The presence of these substances in the environment holds significance not only from an environmental standpoint but also as a means of gathering valuable societal insights. Wastewater analysis, in particular, serves as a powerful tool for monitoring trends within society over the long term, offering insights into drug usage patterns, prevalence rates, and the scale of substance abuse within communities [7,8,9]. An increased concentration of MET in wastewater signals the presence of illicit drug use and production within a given area.

Efforts to monitor and combat illegal MET production and distribution require innovative approaches and technologies. Traditional methods of law enforcement and interdiction have proven insufficient in stemming the tide of MET proliferation. Therefore, the rapid and reliable identification of MET and its precursors presents significant challenges to law enforcement and forensic laboratories. According to the recent review article [10], the analysis of MET encompasses the following steps: sampling and storage, sample preparation either with or without solid-phase extraction, and quantification primarily using liquid chromatography, coupled with tandem mass spectrometry. Advanced ultra-high-performance liquid chromatography has also been reported [11]. Another possibility is to use nano-scaled covalent organic framework-based microspheres as effective sorbents of MET from wastewater with further chromatographic analysis [12]. These methods provide accurate results but are often time-consuming, labor-intensive, and expensive. Furthermore, the evolving nature of synthetic drug production necessitates the development of rapid and portable analytical devices capable of detecting trace amounts of MET [13,14]. Computer-assisted design combined with 3D printing [15] enabled the development of tailored, cost-effective electrochemical detection platforms, leveraged in recent years [16,17,18,19]. These innovative solutions offer the potential for real-time monitoring of environmental samples and urban settings. Compared to conventional separation methods, electrochemical sensors represent potential alternatives to overcome the limitations of traditional techniques, such as fast response, low maintenance, and low cost. However, the development of multi-layered biosensors is often complicated, requires several operations, and is not suitable for field use, especially in the sewage system [20].

Electrochemical techniques were employed to sense MET in various real matrices, including seized samples/tablets, beverages, street samples, liquid aerosols, scout cases, household surfaces, blood/plasma/serum, urine, saliva, and sweat [21,22,23,24,25,26,27,28,29,30,31,32]. To the best of our knowledge, there are only a few works utilizing electrochemical methods for sensing MET or related derivatives in wastewater. Yuan et al. developed the electrochemical aptasensor based on Au nanoparticles/carbon dots/chitosan nanocomposite on a planar gold electrode and demonstrated its feasibility for MET sensing in drinking water, river water, lake water, and untreated wastewater [33]. Truta et al. presented the electrochemical sensor for MET based on a screen-printed carbon working electrode modified with graphene and showed its functionality for confiscated street samples, tap water, and wastewater samples [34]. Dragan et al. performed a comprehensive study aimed at finding electrochemical fingerprints of illicit drugs, including 3,4-methylendioxymethamfetamine, on various carbon-based as well as noble metal electrodes. Obtained characteristics were applied to successfully determine these drugs on graphene and multi-walled carbon nanotubes as the best-performing electrode materials in tap water and wastewater [35]. De Rycke et al. developed a multiplex capacitive sensor based on molecularly imprinted polymers to sense amphetamine and its synthetic precursors, N-formyl amphetamine and benzyl methyl ketone, in sewage water [36]. In a related work, El-Akaad et al. developed a molecularly imprinted polymer-based capacitive sensor for 4-methyl-5-phenylpyrimidine, a marker for the illicit production of amphetamine in wastewater [37]. All the above works succeeded at sensing MET or related compounds under quiescent conditions. We are not aware of any contribution reporting on the electrochemical sensing of these compounds in flowing media, which is critical for their monitoring under real conditions of sewage systems.

To bridge this knowledge and technological gap, here we develop and present a novel electrochemical technique for the determination and monitoring of MET and its precursor APAAN in quiescent as well as flowing wastewater samples at controlled laboratory conditions. To this end, we design and manufacture a dedicated 3D-printed (3DP) cell prototype, with an embedded screen-printed chip with a carbon-based working electrode. The results of our study serve as a springboard for further modifications of 3DP cells as models for future use in real sewage systems. The primary objective is to demonstrate the analytical feasibility of direct electrochemical sensing of MET (and APAAN) in a real wastewater matrix under controlled flow, using a low-cost 3DP flow-through cell as a laboratory proxy for sewer hydraulics.

The necessity for a device that is easily operable, portable, and durable, in conjunction with a reliable chip capable of verifying or refuting suspected illegal manufacturing, constitutes a crucial element of our paper. Despite the simplicity of the chip’s design, the more complex sandwich-type sensors described in the literature review appear to be inappropriate in the aggressive wastewater environment. Associated with this is the development of laboratory-scale proof-of-concept that could serve as a model to predict the suitability of the analytical method for direct MET detection in real wastewater under controlled conditions.

## 2. Materials and Methods

### 2.1. Manufacturing of 3DP Flow Cell

**Figure 1:**
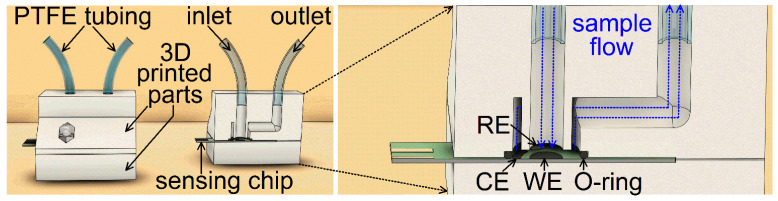
(**left**) Full and cross-sectional image of the cell designed and 3DP in this work. (**right**) Details of the channels and the screen-printed chip.

The 3DP cell (Figure 1) developed in this work is derived from our previous designs tailored to electrochemical sensing and related applications [38,39,40]. The current cell design enables analyte sensing in static as well as controlled-flow arrangements and is compatible with commercial chips with screen-printed electrodes. The cell was devised in Autodesk Fusion 360 (Autodesk Inc., San Francisco, CA, USA) computer-assisted design (CAD) software (version 2605.1.52). The resulting geometry was exported as a high-resolution .stl file to PrusaSlicer (version 2.0.0), where instructions for the 3D printer were programmed. The cell consists of two parts connected by a pair of steel nuts and bolts. The lower part of the cell has a cutout to place the chip (Figure 1, right). An O-ring mounted to a customized groove in the upper part of the cell is used to prevent the leaking of introduced samples. The upper part of the cell further contains a system of sample flow channels. The sample is introduced to the inlet channel via a PTFE tube using a peristaltic pump PCD 1081 (Čerpadla Kouřil, Kyjov, Czech Republic). The inlet channel directs the sample towards the working electrode (Figure 1, left) of the same diameter as the inlet channel. The sample is drained by a circular shaft and leaves the cell through a side outlet channel.

Cell parts were manufactured employing an extrusion-based Prusa I3 MK3 printer equipped with a brass extrusion nozzle (0.4 mm diameter) and smooth polyetherimide-coated steel spring sheet printing pad (all items purchased from Prusa Research, Czech Republic). Transparent PLA filament (Gembird, Almere Haven, The Netherlands) was utilized as the printing material. Just before printing, the extruder was copiously purged with this filament at 250 °C to remove residues of previously used materials. The printing pad was cleaned with acetone (Petr Švec-PENTA s.r.o., Prague, Czech Republic) at ambient temperature. No agents modifying the adhesion between printed objects and the printing pad were utilized. The bottom part of the cell was printed in the bottom-up direction, while the top part was printed in the top-down direction. The printing layer height was set to 0.1 mm, and the extrusion multiplier to unity. The temperature of the printing pad and the extrusion nozzle was 60 °C and 225 °C, respectively. The printing speed values were set as follows: first layer 20 mm s^−1^, external perimeters 25 mm s^−1^, internal perimeters 45 mm s^−1^, infill 80 mm s^−1^, and non-printing moves 180 mm s^−1^. Retraction settings were set to default values for PLA. Cell parts have internal and external shells composed of two perimeters and 100% infill (complete solidity). An external brim with a width of 10 mm was added to the first layer of cell parts to improve their adhesion to the pad during printing. No material support was employed. Upon printing, cell parts were left to cool down to the ambient temperature and were gently detached from the pad with a scalpel blade. The brim was removed with scissors. Cell parts were stored in closed glass vessels to minimize ambient contamination.

### 2.2. Electrochemical Measurements in the 3DP Flow Cell

The activation of the carbon working electrode (WE) with a diameter of 4 mm of the screen-printed chip (purchased from Metrohm Česká republika s.r.o., Prague, Czech Republic) was performed directly in the developed 3DP cell filled with aqueous 0.1 M KCl (ACS reagent grade, Sigma Aldrich) solution, which served as the activation electrolyte. The screen-printed chip integrates an Ag/AgCl quasi-reference electrode (RE) and a carbon counter electrode (CE). First, an electrochemical impedance spectrum was recorded at 0.0 V DC potential vs. the RE employing an AC waveform with peak-to-peak amplitude of 10 mV and frequency ranging from 100 to 10^5^ Hz. The resistance between the WE and the RE was extracted from the high-frequency part of the spectrum and served as the input parameter for positive feedback electronics to compensate for Ohmic voltage losses during the activation procedure, as well as in subsequent characterization of activated WE by cyclic voltammetry (CV) (vide infra). For the activation, the WE was polarized at −2.0 V vs. RE for 180 s and subsequently at +2.0 V for the same duration. Upon activation, the cell was copiously purged with fresh 0.1 M KCl solution, additionally containing 2 mM [Ru(NH_3_)_6_]Cl_3_ as the standard redox probe (98%, Merck, Bratislava, Slovakia). CV was performed at various WE polarization rates ranging from 0.01 to 0.50 V s^−1^ employing a linear scan module within the accessible potential window of the electrode/electrolyte interface under no-flow and flow conditions. All electrochemical measurements in this work were performed 3 times employing an Autolab potentiostat (Metrohm, Česká republika s.r.o., Prague, Czech Republic). Oxygen was intentionally not removed from inspected samples to mimic real wastewater conditions, where naturally dissolved oxygen is present.

### 2.3. MET Determination in Wastewater in the 3DP Flow Cell

Wastewater used as the matrix for MET determination was taken from the influent of the municipal wastewater treatment plant (WWTP), and before the experiment, it was filtered through a 0.45 µm pore size hydrophilic syringe filter (Merck, Bratislava, Slovakia). The stock solution of MET obtained from police intervention was diluted in deionized water to a 10 mg L^−1^ concentration and spiked into the filtered wastewater. These MET solutions were prepared by the laboratories of the Criminalistics and Expertise Institute of the Police Force of the Slovak Republic. Square-wave voltammetry (SWV) and chronoamperometry at optimized conditions were used as detection techniques in both flow and non-flow arrangements [41]. In our workflow, one chip was used for a single calibration/experiment series and then replaced; under real wastewater the chip could be used for ~15 consecutive measurements before signal quality degraded. SWV was performed using a frequency of 60 Hz, at an amplitude of 50 mV in the potential range from 0 V to 1.2 V vs. an Ag/AgCl quasi-RE on the chip to perform measurements as easily and robustly as possible. Chronoamperometry was performed at WE potentials corresponding to diffusion-limited MET oxidation.

### 2.4. Sensor Specificity Towards APAAN and Amphetamine and Simulated Sewage Tests

Simultaneous detection of α-phenylacetoacetonitrile (APAAN), amphetamine (AMP), and MET in wastewater is crucial for understanding and combating synthetic drug abuse, tracing clandestine production networks, and protecting environmental health. To test sensor specificity, a precursor of amphetamine-like drug production, APAAN and AMP, both provided by the Criminalistics and Expertise Institute of the Police Force of the Slovak Republic, were dissolved in methanol and spiked into filtered wastewater (both at the same concentration as for MET detection). SWV was used as the detection technique in a flow arrangement under the same conditions as for MET determination (see above). These tests were carried out using the same 3DP cell with a flow rate of 3.5 mL s^−1^, employing the same wastewater as in the case of MET.

## 3. Results

### 3.1. Cell Characterization

**Figure 2:**
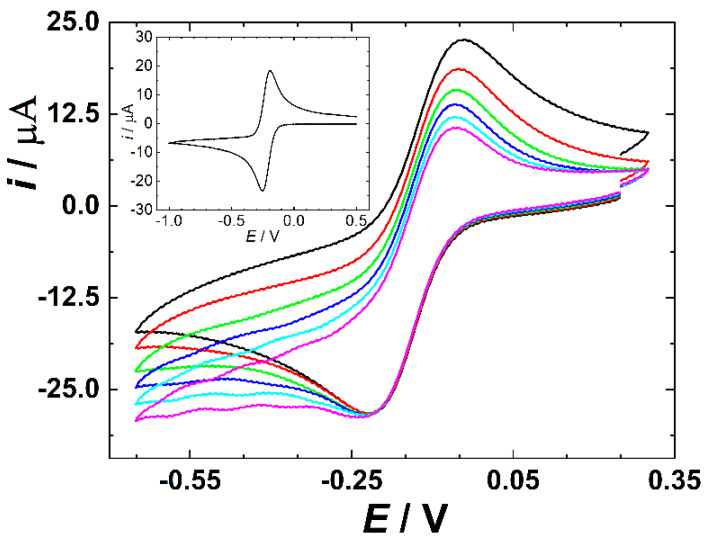
CV scans of 2 mM [Ru(NH_3_)_6_]Cl_3_ in 3DP cell at various flow rates (from black to pink curve: 0.1; 0.7; 1.4; 2.1; 2.8; 3.5 mL s^−1^); scan rate of 0.1 V s^−1^. Inset: CV obtained under no flow conditions at 0.1 V s^−1^.

Before determining the aforementioned analytes in spiked wastewater, the functionality of the developed 3DP flow cell was first inspected by evaluating the cyclic voltametric (CV) response of [Ru(NH_3_)_6_]Cl_3_ standard redox probe (additionally containing 0.1 M KCl as the supporting electrolyte) under stationary conditions (Figure 2, inset). Both cathodic and anodic potential scans show a well-evolved faradaic current maximum corresponding to the reduction and subsequent re-oxidation of the probe, respectively. The peak-to-peak separation of the cathodic and anodic faradaic current maxima amounts to 63 ± 3 mV, being very close to the theoretical prediction for the diffusion-limited reversible transfer of one electron (59 mV). This indicates that the surface of the activated WE has almost no kinetic hindrance for the interfacial charge transfer. Subsequently, the CV analysis was repeated under flow conditions with systematically varied sample flow rate (main panel of Figure 2).

Similar to the stationary conditions, both anodic and cathodic voltage scans show well-developed faradaic maxima corresponding to the reduction/oxidation of the redox probe. Sample flow seems to have, under selected conditions, no measurable influence on the cathodic faradaic peak current magnitude, but slightly increases currents measured at more negative potentials, which we ascribe to the augmented supply of depleted reactant by the convective mass transport. Similarly, the effect of the sample flow on the anodic faradaic response is minimal. We conclude that the cyclic voltametric response of the redox probe retains its basic characteristics under selected flow conditions, making the voltammetry a suitable tool for sensing target analytes in the developed 3DP cell. Moreover, at a flow rate of 3.5 mL s^−1^ and channel diameter of approximately 4 mm, the mean velocity is ~0.28 m s^−1^, i.e., comparable to commonly used minimum design velocities (~0.3 m s^−1^) and below typical self-cleansing peak velocities (~0.6 m s^−1^) used in real sewage systems.

### 3.2. Determination of Methamphetamine in 3DP Flow Cell

As the next step, the developed 3DP cell equipped with a commercial screen-printed chip with a carbon WE was applied to determine MET in wastewater employing the SWV regime. The mechanism of MET oxidation underlying the faradaic response as the analytically useful signal is well described in the literature [34,42].

**Figure 3:**
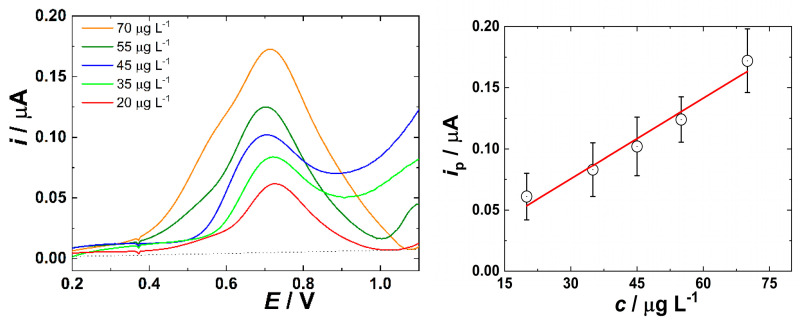
(**left**) SWVs of wastewater spiked with MET obtained in the 3DP flow cell developed in this work under stationary conditions after subtracting the curve measured without MET (background). (**right**) Dependence of faradaic peak current on MET concentration after background correction. SWV was performed using a frequency of 60 Hz, at an amplitude of 50 mV. Error bars represent the standard deviation across 3 independent replicate measurements.

First, measurements were performed in the non-flow arrangement with results shown in Figure 3, left. The pH of the wastewater taken from the influent of WWTP was adjusted from 7.5 to 9.5 to increase the sensitivity of MET determination [43]. The oxidation peak is well-developed, and a nearly linear dependence of the faradaic peak current on the increasing concentration is observed (Figure 3, right).

Compared to previous studies, the determination of MET in filtered wastewater is more demanding and complicated than in a simple model buffer [34,44]. Oxidation peaks in wastewater are not as symmetric as in the model solution, and the error bars representing standard deviation among individual SWV analyses reach from 10 to 30% (right panel of Figure 3). The limit of detection LOD = 15.9 µg L^−1^ was calculated according to the equation LOD = (3.3 × σ)/k, where σ is the standard deviation of the response and k is the slope of the calibration curve. This higher value of LOD compared to the previous works in the model buffered solutions may be caused by the presence of various biomolecules and organic compounds (proteins, fatty acids, saccharides and polysaccharides, nucleic acids or their residues, micropollutants and their metabolites, etc.) in real wastewater [1,7], potentially adsorbing on the WE surface of the chip and complicating the analyte sensing at its ultralow concentrations as examined in this work. In particular, adsorbed high molecular weight biomolecules can decrease the electrochemically active surface area of the WE or retard charge transfer kinetics, in both cases increasing LOD.

**Figure 4:**
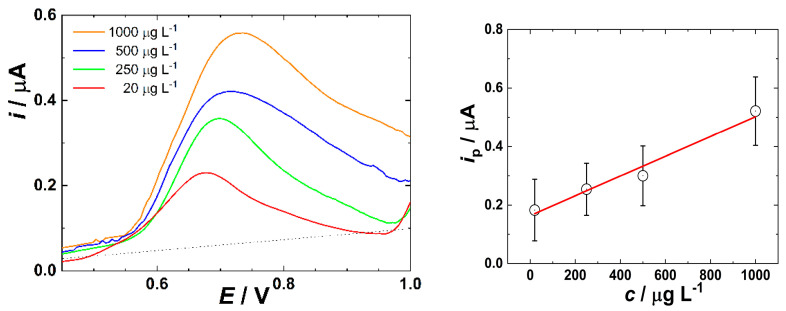
(**left**) SWVs of wastewater spiked with MET obtained in the 3DP flow cell developed in this work under flow conditions (sample flow rate of 3.5 mL s^−1^) after subtracting the curve measured without MET (background). (**right**) Dependence of faradaic peak current on MET concentration after subtracting the curve measured without MET (background). SWV was performed using a frequency of 60 Hz, at an amplitude of 50 mV. Error bars represent the standard deviation across 3 independent replicate measurements.

The abovementioned complication may become even more pronounced in the flow arrangement, reflecting real environmental conditions in a sewage system (Figure 4). One key challenge was the inability to detect MET at the low concentrations achievable under non-flow conditions [42,45,46], with the sensing being applicable only for higher MET concentrations. Additionally, in our work, the pH was intentionally not adjusted to the value of 9.5 to fulfill the optimized conditions for MET determination, as such modifications are not feasible in real flowing sewage systems.

Under the flow regime, the SWV response of MET is less developed (Figure 4, left) compared to the observations reported in the literature [47] and findings obtained in this work under non-flow conditions (Figure 3, left). Also, oxidation peak potential slightly shifts (ca by 50 mV) to more positive values with the increasing MET concentration. Presumably, such behaviour results from the combination of the irreversible nature of MET oxidation and/or increased charge transfer resistance due to (bio)fouling of the WE, facilitated by the convective flow [48]. Interestingly, at the lowest tested MET concentration (20 µg L^−1^), the absolute SWV current response in flow was approximately four times higher than that recorded under non-flow conditions. This suggests that convective mass transport initially enhances analyte delivery to the electrode surface, leading to a stronger current response, particularly at low concentrations. However, this apparent advantage does not translate to better analytical performance across the entire concentration range. Indeed, calibration slope values, representing the analytical sensitivity of the system, reveal a contrasting trend. The slope for the flow regime was nearly one order of magnitude lower than the slope under non-flow conditions. This reduction in sensitivity is also most likely attributable to enhanced adsorption of interfering organic and inorganic substances from the wastewater matrix, which increasingly block the active surface of the carbon-based electrode during prolonged exposure in flow. The dynamic fouling under convective transport thus appears to override the initial benefit of enhanced mass transfer, particularly at higher concentrations where matrix effects are more pronounced.

A further factor contributing to the reduced performance under flow conditions is the difference in pH between the two regimes. In the non-flow arrangement, the wastewater pH was adjusted to 9.5, whereas in the flow regime, no pH adjustment was made, and the natural value remained at approximately 7.5. The electrochemical activity of the secondary amino group in MET is significantly enhanced under basic conditions, which correlates with its pKa value of 10.1. Therefore, the more favorable oxidation behavior observed in the stationary regime can be partially explained by the increased deprotonation of the amino group at higher pH, leading to improved electron transfer kinetics. Taken together, these observations illustrate a complex interplay between convective transport, matrix fouling, and solution pH, all of which critically influence electrochemical detection of MET in real wastewater. The LOD of 211.2 µg L^−1^ determined by SWV in the flowing regime is approximately 13 times higher compared to the non-flow conditions (15.9 µg L^−1^) and more than 2 orders of magnitude higher compared to the ideally buffered quiescent solution [43].

An important aspect of this study concerns the potential of electrochemical techniques for continuous monitoring of MET in real flowing sewage systems. In particular, chronoamperometric (CA) techniques are commonly employed due to their operational simplicity, fast response, and compatibility with remote data collection platforms. In flowing media applications, the sensor chip remains submerged within the wastewater stream, continuously recording current response at a fixed applied WE potential corresponding to the oxidation (or reduction) of the sensed species, with obtained data transmitted and evaluated remotely, enabling unattended and real-time monitoring. To simulate these conditions, the CA sensing of MET was conducted using the developed 3DP flow cell. The WE potential was set to 0.75 V, i.e., corresponding to the diffusion-limited MET oxidation (see Figure 3, left and Figure 4, left) under the flow regime. In the absence of MET in the solution, a stable background current was recorded. Upon introducing the solution of MET to the cell, a marked increase in current was observed corresponding to MET oxidation. Upon subsequent injection of MET-free solution (pure wastewater), the current signal returned to the baseline level, confirming that the system can detect the transient presence of MET under flow conditions, which simulates real sewage monitoring scenarios.

However, the use of CA in complex wastewater matrices presents several limitations. A primary concern is the elevated background current, particularly under convective flow, which can obscure analyte-specific signals. Such parasitic response may originate from a broad range of electroactive substances naturally present in wastewater, including pharmaceuticals, metabolites, and biomolecules. In the absence of an upstream separation technique (e.g., chromatographic pretreatment), the measured faradaic signal represents summed contributions of all present oxidizable species, making it difficult to attribute the signal exclusively to MET. Furthermore, prolonged sensor exposure to wastewater leads to WE surface (bio)fouling, primarily due to the adsorption of high molecular weight organic compounds and biomolecules such as humic substances, proteins, polysaccharides, and microbial residues. These (bio)foulants progressively alter the electrochemical sensing interface, reduce its active surface area, and unpredictably degrade the sensitivity over time [49], which is consistent with the observations and with prior studies on carbon electrodes in complex matrices.

While this study intentionally employed a simple, unmodified screen-printed chip with a carbon WE to preserve the practicality, affordability, and disposability of the system, (bio)fouling remains a critical challenge for real sewage applications. To address this, future work could explore the integration of periodic electrochemical cleaning (regeneration) protocols, such as anodic or cathodic steps or sweep potential excursions, which have been shown to desorb adsorbed contaminants and partially restore electrode sensitivity without manual intervention [50]. This approach is particularly suitable for flow systems, where automated pulse cycles could be incorporated into the measurement protocol to prolong sensor lifespan and improve data consistency.

### 3.3. Chip Selectivity Tests

**Figure 5:**
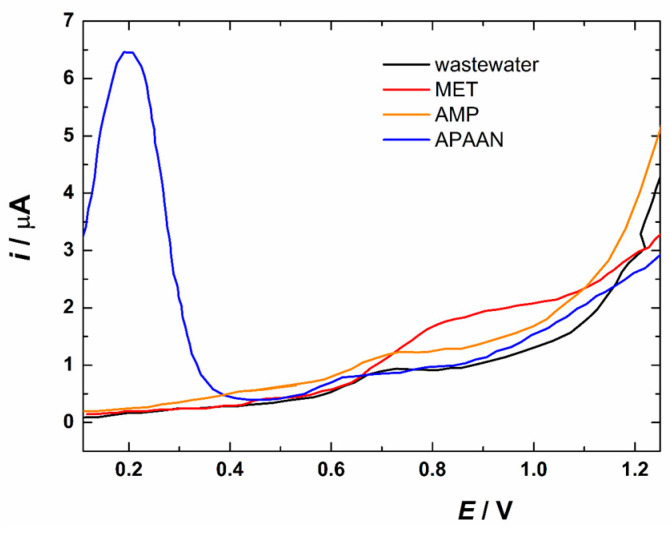
SWV response of filtered wastewater spiked with APAAN, AMP, and MET at a concentration of 1.5 mg L^−1^. The black curve shows the baseline response (non-spiked filtered wastewater). All conditions of SWV are the same as for MET detection in 3DP cell under the flow conditions (3.5 mL s^−1^).

Detecting MET alone, however, is not sufficient to fully understand and combat the broader issue of synthetic drug abuse. APAAN serves as a critical precursor in the synthesis of illicit amphetamine-type stimulants, including MET. Monitoring the presence of APAAN in conjunction with MET can provide valuable insights into the scale and operational methods of clandestine drug manufacturing networks. Moreover, AMP, both a precursor and a final product of various synthetic routes, has its own set of abuse and health implications. Therefore, the capability of SWV sensing of MET simultaneously with APAAN and AMP was also examined in this work (Figure 5). For APAAN (blue curve), a strong faradaic response was detected at a potential of +0.20 V, while only a subtle signal was found for AMP (orange curve), centered around 0.70 V. Clearly, these responses do not interfere with the sensing of MET (oxidation potential around 0.80 V, red curve). The response obtained for AMP (orange curve) is very similar to the baseline response recorded in the non-spiked filtered wastewater (black curve), indicating that AMP was not detected by the developed 3DP flow-through system under the given experimental conditions. APAAN contains aromatic and carbonyl/nitrile functional groups that have more pronounced and sharper redox signals in the accessible potential window of the electrode/sample interface, thus giving a clear electrochemical signature. On the other hand, the primary/ammonium groups in amphetamines are protonated at common (neutral/acidic) pH; the protonated form is more difficult to oxidize, so oxidation of amphetamines often requires higher (or specific) potentials and/or alkaline conditions. In practice, this means that without special modifications or derivatization, some pre-precursors (e.g., APAAN) will be measurable faster and with less interference than the amphetamine compounds themselves. We conclude that well-separated and distinguishable signals obtained for MET and APAAN (Figure 5) allow for side-by-side detection of these substances in wastewater. A complementary approach to monitoring amphetamine-type synthetic drugs and their precursors, based on the concept of electrochemical fingerprints (i.e., comparison of voltammetric profiles), has already been demonstrated under optimized laboratory conditions (using an appropriate supporting electrolyte) in confiscated samples [44]. It would be particularly interesting to explore whether a similar principle could be applied to real wastewater samples.

## 4. Discussion

The growing demand for rapid, in situ detection of illicit drugs and their precursors in wastewater highlights the need for simple, sensitive, selective, robust, affordable, and easily deployable sensing platforms. Real-time monitoring has particular value in scenarios such as police interventions, forensic investigations, and the surveillance of areas suspected of harboring clandestine drug laboratories. In such applications, rapid decision-making and on-site detection are essential, requiring devices that are not only sensitive and selective but also operationally straightforward and resistant to matrix-related interferences.

This study demonstrates that sensitive and selective electrochemical MET detection can be achieved in flowing wastewater using a dedicated 3DP flow-through cell equipped with an unmodified, commercially available chip with a carbon-based WE. This is particularly advantageous as an actionable foundation for future real-time field deployment as it eliminates the need for complicated WE modifications or multilayered biosensor designs, which are often impractical in real-world settings. In the context of wastewater, which contains a wide range of organic and inorganic potentially interfering substances, the simplicity and disposability of the sensor are critical. Modified sensors, while often highly selective in controlled environments, tend to degrade rapidly or become fouled in complex matrices such as wastewater, reducing their reliability for continuous use. The advancement of this study lies primarily in the application scenario (direct sensing in real wastewater under flow using an unmodified disposable SPCE integrated into a 3DP flow-through platform), rather than achieving the lowest possible LOD under ideal buffered/quiescent conditions. This is compared in Table 1 below.

One of the most important findings of this study is the ability of the sensor to distinguish MET from structurally and synthetically related substances (mainly APAAN). This side-by-side detection capacity supports the concept of using the platform in police operations or forensic screening, where rapid differentiation between drug components is needed. The clear signal separation without the need for sample pretreatment or WE functionalization presents a key advantage for rapid assessments in the field.

However, the limitations of using simple sensors in real wastewater must be admitted. The complexity of the wastewater matrix, consisting of humic substances, suspended solids, surfactants, and high-molecular-weight organic compounds, can lead to sensor (bio)fouling and gradual loss of faradaic response as the analytical signal, especially during prolonged measurements or continuous monitoring [52]. These effects are evident in this work in the deterioration of the MET detection limit in the flow regime, i.e., being applicable for reliable detection of MET only at elevated concentrations. In addition, pH variability in wastewater, which cannot be easily controlled or adjusted in field conditions, affects the faradaic response of target analytes and can reduce the analysis reproducibility and sensitivity.

To address these challenges, future developments could focus on improving sensor durability and matrix tolerance, potentially through low-cost antifouling layers [53,54] or self-cleaning mechanisms (such as the application of periodic anodic or cathodic cleaning pulses, which could help desorb accumulated contaminants from the WE surface and fully or at least partially restore the sensor performance) compatible with unmodified Wes [55,56]. Furthermore, data processing algorithms capable of correcting for baseline drift and noise induced by fluctuations of wastewater flow and composition could enhance the reliability of results without increasing hardware complexity [57,58].

The integration of such simple sensors into mobile or stationary monitoring units, including sewer-mounted probes or vehicle-based systems used during police operations, could revolutionize illicit drug detection. Real-time data transmission to centralized systems would allow authorities to map drug activity hotspots, estimate the scale of production or consumption, and respond more rapidly to emerging threats. Such systems could also contribute significantly to wastewater-based epidemiology, providing near-instantaneous insights into drug trends at the community level.

## 5. Conclusions

Illicit drug abuse is a significant global problem, with widespread health, social, and economic consequences. Monitoring the prevalence of drug use in a population is crucial for developing effective prevention and treatment strategies. One approach to addressing this challenge is analyzing wastewater, which can provide valuable insights into the consumption patterns of the local community.

In this study, we present the development and application of a sensitive, selective, robust, affordable, and easily deployable electrochemical method for the determination of MET as a representative illicit drug in quiescent as well as flowing wastewater media. To achieve this, we designed and manufactured a 3DP flow-through cell with an embedded commercial sensing chip, allowing direct electrochemical detection of MET in wastewater samples, fulfilling all the above-listed demands.

The cell was devised using computer-aided design software and printed using a desktop extrusion-based 3D printer. The functionality of the cell was verified under controlled laboratory conditions using a standard redox probe in the non-flow and flow arrangements. At the beginning, the cell was tested in a controlled environment. Subsequently, the cell was applied for MET determination in the filtered flowing wastewater, with the achieved detection limit of 211.2 µg L^−1^. Besides high sensitivity, the sensor showed high specificity for MET, as demonstrated by a signal distinguishable from that of its synthetic precursor APAAN.

By simultaneously detecting APAAN and MET in flowing wastewater, law enforcement and public health agencies can gain an immediate and comprehensive understanding of the synthetic drug landscape. The aim of this work was not solely the construction of a highly sensitive chip for continuous monitoring of a particular environmentally relevant substance, but also to explore the possibility of manufacturing microfluidic sensing devices using extrusion 3D printing, a technique that we additionally consider to have immense potential for the rapid prototyping of models mimicking a real municipal sewage system. All our results confirm that the 3DP cell designed and manufactured in this research can be successfully used as a small-scale laboratory model for determining environmentally relevant analytes directly in flowing sewage systems. This capability provides valuable insights into drug usage patterns and aids in the detection of illicit drug production.

In summary, the developed 3DP flow-through cell provides a pragmatic foundation for near real-time monitoring of MET and related synthesis markers directly in flowing wastewater. Its manufacturability on desktop printers, compatibility with off-the-shelf electrodes, and ability to differentiate target analytes under flow conditions recommend it for translation to mobile or fixed monitoring units supporting forensic, environmental, and public health decision-making. Future work will focus on antifouling and auto-cleaning protocols, long-term stability studies, and integration with remote telemetry to enable scalable sewer surveillance networks.

## Figures and Tables

**Table 1:** Comparison of representative electrochemical methods for methamphetamine detection in water samples.

Electrode Type/ Modification	Matrix	Sample Pretreatment	Operating Regime	LOD	Key Constraints	Ref.
SPCE	Real wastewater	Filtration	Flow Static	15.9 µg L^−1^ 211.2 µg L^−1^	Matrix fouling, lower sensitivity in flow, realistic conditions	This work
Au@carbon dots/chitosan nanocomposite modified aptasensor	Drinking water, river, lake, wastewater	None	Static	0.87 pg L^−1^	More complex biorecognition	[33]
Graphene-modified SPCE	Tap water, wastewater	Diluted with PB	Static	300 nM	Needs electrode modification	[34]
MWCNTs graphite electrode	Wastewater	Diluted with PB	Static	Not reported	Needs electrode modification	[35]
Boron-doped diamond	Phosphate buffer	None	Static	0.08 µg L^−1^	Ideal lab matrix, not wastewater-applicable	[43]
Laser-induced porous graphene electrode	BR buffer	None	Static	0.31 µg mL^−1^	Need for electrode preparation	[46]
Aptamer/AuNPs/Chitosan/GCE	Phosphate buffer	None	Static	10 nM	Long sensor preparation	[51]

SPCE—Screen-printed carbon electrode; MWCNTs—Multi-walled carbon nanotubes; AuNPs—gold nanoparticles; GCE—glassy carbon electrode; PB—phosphate buffer; BR—Britton-Robinson buffer.

## Data Availability

The data supporting the findings of this study are available from the corresponding author upon reasonable request. Due to the involvement of law enforcement and the sensitive nature of some data related to police-supplied materials, certain datasets are not publicly available for legal and ethical reasons.
